# Identification of Plasma Metabolomic Biomarkers of Juvenile Idiopathic Arthritis

**DOI:** 10.3390/metabo14090499

**Published:** 2024-09-16

**Authors:** Amar Kumar, Joshua Tatarian, Valentina Shakhnovich, Rachel L. Chevalier, Marc Sudman, Daniel J. Lovell, Susan D. Thompson, Mara L. Becker, Ryan S. Funk

**Affiliations:** 1 Center for Computational Biology, University of Kansas, Lawrence, KS 66047, USA; 2 University of Kansas School of Medicine, Kansas City, KS 66160, USA; 3 Ironwood Pharmaceuticals, Boston, MA 02110, USA; 4 University of Missouri-Kansas City School of Medicine & Children’s Mercy Kansas City, Kansas City, MO 64108, USA; 5 Cincinnati Children’s Hospital Medical Center, Cincinnati, OH 45229, USA; 6 Division of Rheumatology, Department of Pediatrics, Duke University School of Medicine, Durham, NC 27710, USA

**Keywords:** autoimmune disease, juvenile idiopathic arthritis, metabolomics, biomarkers, disease-modifying antirheumatic drugs

## Abstract

Identification of disease and therapeutic biomarkers remains a significant challenge in the early diagnosis and effective treatment of juvenile idiopathic arthritis (JIA). In this study, plasma metabolomic profiling was conducted to identify disease-related metabolic biomarkers associated with JIA. Plasma samples from treatment-naïve JIA patients and non-JIA reference patients underwent global metabolomic profiling across discovery (60 JIA, 60 non-JIA) and replication (49 JIA, 38 non-JIA) cohorts. Univariate analysis identified significant metabolites (*q*-value ≤ 0.05), followed by enrichment analysis using ChemRICH and metabolic network mapping with MetaMapp and Cytoscape. Receiver operating characteristic (ROC) analysis determined the top discriminating biomarkers based on area under the curve (AUC) values. A total of over 800 metabolites were measured, consisting of 714 known and 155 unknown compounds. In the discovery cohort, 587 metabolites were significantly altered in JIA patients compared with the reference population (*q* < 0.05). In the replication cohort, 288 metabolites were significantly altered, with 78 overlapping metabolites demonstrating the same directional change in both cohorts. JIA was associated with a notable increase in plasma levels of sphingosine metabolites and fatty acid ethanolamides and decreased plasma levels of sarcosine, iminodiacetate, and the unknown metabolite X-12462. Chemical enrichment analysis identified cycloparaffins in the form of naproxen and its metabolites, unsaturated lysophospholipids, saturated phosphatidylcholines, sphingomyelins, ethanolamines, and saturated ceramides as the top discriminating biochemical clusters. ROC curve analysis identified 11 metabolites classified as highly discriminatory based on an AUC > 0.90, with the top discriminating metabolite being sphinganine-1-phosphate (AUC = 0.98). This study identifies specific metabolic changes in JIA, particularly within sphingosine metabolism, through both discovery and replication cohorts. Plasma metabolomic profiling shows promise in pinpointing JIA-specific biomarkers, differentiating them from those in healthy controls and Crohn’s disease, which may improve diagnosis and treatment.

## 1. Introduction

Juvenile idiopathic arthritis (JIA) is an immunoinflammatory form of arthritis in children with notably heterogeneous clinical expression. The disease is mediated through dysregulated immune responses, particularly involving autoreactive T-cells targeting cartilage antigens, leading to inflammation. This imbalance disrupts immune tolerance to self-antigens, resulting in synovial inflammation [1]. Globally, an estimated 3 million children and young adults are affected by JIA, with prevalence rates consistently skewed towards the female population [2].

Unlike adult rheumatoid arthritis (RA), clinicians cannot rely on biomarkers for diagnosing JIA [1]. This shortfall impacts the ability to classify JIA effectively and initiate timely treatment. Although both conditions have been the subject of biomarker exploration, their distinct disease characteristics may impact the utility of uniform biomarkers across the two rheumatologic conditions in the clinical setting.

To detect biomarkers in autoimmune diseases like RA and JIA, a combination of traditional diagnostic methods (e.g., ELISA, Indirect Immunofluorescence, Western blotting) and novel approaches (e.g., biosensors) is employed for more rapid and cost-effective analysis [3]. Recent advancements in omics technologies, such as genomics, proteomics, metabolomics, and transcriptomics, provide comprehensive insights into the molecular alterations underlying these diseases [4]. In RA, key biomarkers include autoantibodies like Rheumatoid Factor (RF), Anti-Citrullinated Protein Antibodies (ACPAs), and Anti-Carbamylated Protein Antibodies (anti-CarP), as well as inflammatory markers (e.g., C-reactive protein (CRP), erythrocyte sedimentation rate (ESR)) and RA-associated genetic factors, such as specific Human Leukocyte Antigen-DR Beta 1 (HLA-DRB1) alleles [5,6,7]. In contrast, JIA shows a distinct biomarker profile with cytokines like Interleukin-1 (IL-1), Interleukin-6 (IL-6), and Interleukin-18 (IL-18) playing a more significant role, particularly in systemic JIA, and with unique Human Leukocyte Antigen (HLA) associations depending on the subtype [7,8,9,10,11,12,13,14]. The pathophysiology of RA is primarily driven by adaptive immune responses, while JIA involves a mix of autoinflammatory and autoimmune mechanisms [15,16,17,18,19].

While some researchers have informally examined diverse biomarkers in JIA and conducted more systematic studies in RA patients, findings from RA studies might not translate seamlessly to JIA, given the inherent differences between the diseases and the age difference across the two distinct patient populations [1,20]. It is critical to account for these differences as age can significantly influence various clinical outcomes, including disease progression, treatment response, and overall prognosis [20]. In addition to early disease diagnosis, the identification of disease biomarkers could improve understanding of disease classification, progression, prognosis, and treatment response [21]. For example, despite the wide use of disease-modifying antirheumatic drugs (DMARDs) like methotrexate (MTX) for JIA and RA [22,23], the response to DMARD treatment is highly variable, resulting in a trial-and-error approach. Thus, the identification of reliable diagnostic and therapeutic biomarkers is imperative for aiding clinicians in clinical decision-making. Such biomarkers would enable more accurate and individualized dosing early in the disease process, thereby minimizing the need for subsequent dose adjustments.

Research highlights the role of metabolites in JIA pathogenesis, including microbial metabolites like Short-Chain Fatty Acids (SCFAs) that contribute to inflammation through gut–joint interactions, plasma metabolites like furaneol sulfate mediating gut microbiota–JIA relationships, and serum metabolites such as kynurenine and 3-dehydrocarnitine, which are causally linked to JIA and involved in key metabolic pathways driving disease onset and progression [24,25,26]. Machine learning models and bioinformatics further enhance biomarker identification by analyzing miRNA sequences, offering improved diagnostic accuracy, personalized treatment, and better disease prediction, thus shaping the future of biomarker discovery in autoimmune diseases [27,28].

Metabolites are byproducts or intermediates of enzyme activity and exogenous exposures. They serve as a surrogate for an organism’s metabolic activity and environmental exposure and can depict its phenotype at a given time. We evaluated plasma metabolomic profiles in treatment-naïve pediatric patients with JIA compared to the following two types of controls: those without autoimmune disorders and those with another chronic autoimmune condition (Crohn’s disease).

## 2. Materials and Methods

### 2.1. Patients

For this study, we utilized plasma samples from patients diagnosed with JIA collected prior to the initiation of disease-modifying antirheumatic drug (DMARD) therapy and plasma samples from a reference non-JIA population of pediatric patients that included children with active Crohn’s disease who had not been exposed to systemic immuno-modulators, immunosuppressants, or biologics, and otherwise healthy, age-matched controls without autoimmune disorders or discernable gastrointestinal or rheumatologic pathology on clinical, laboratory, or histopathological findings. The patient samples were collected from the following centers: Children’s Mercy Kansas City (CM-KC) in Missouri and Cincinnati Children’s Hospital Medical Center (CCHMC) in Ohio. The 60 JIA subjects in the discovery cohort were from the CM-KC IRB-approved MTXR cohort. The 49 JIA subjects in the replication cohort were enrolled through the IRB-approved PROMOTE study in CCHMC and CM-KC. The non-JIA samples (*n* = 98) came from a biorepository (single IRB-approved study) at CM-KC in Missouri. All non-JIA subjects from the CM-KC biorepository were fasted for at least 8 h. The non-JIA subjects chosen were age-matched and confirmed to not have any organic causes for their gastrointestinal (GI) symptoms, including no significant findings on histopathology from tissues biopsied during endoscopy. Patients provided age-appropriate informed consent or assent.

**Figure 1:**
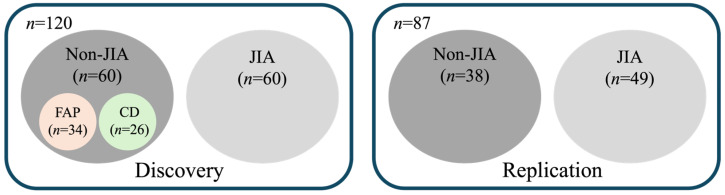
Composition of the discovery and replication cohorts for metabolomic profiling. The discovery cohort includes a total of 120 patients, with 60 representing DMARD-naïve children with JIA and 60 representing non-JIA reference patients. The non-JIA cohort includes subjects with Functional Abdominal Pain (FAP) and Crohn’s disease (CD). The replication cohort includes a total of 87 patients composed of 49 DMARD-naïve children with JIA and 38 non-JIA reference patients.

The patient cohorts underwent separate analyses in two batches as follows: the discovery cohort, comprising 120 samples, and the replication cohort, consisting of 87 samples. The age range for the discovery cohort was 1.75–18.08 years, while the replication cohort spanned from 1.50–20.33 years. Within the replication cohort, there were subgroups of non-JIA (*n* = 38) and JIA (*n* = 49), while the discovery cohort’s non-JIA group (*n* = 60) included samples from patients with Crohn’s disease (*n* = 26) and Functional Abdominal Pain (*n* = 34), alongside the JIA cohort (*n* = 60) (Figure 1). Notably, the non-JIA subgroup in the replication cohort also incorporated samples from a pharmacokinetic study of healthy individuals. In this manuscript, “non-JIA” collectively denotes healthy control subjects from the pharmacokinetic study for the replication cohort and individuals with Crohn’s disease and Functional Abdominal Pain for the discovery cohorts, unless explicitly stated otherwise.

### 2.2. Sample Preparation

Venous blood samples were processed to extract plasma using a Beckman tabletop centrifuge at 2000 RPM for 10 min. Following extraction, the plasma supernatant was divided into aliquots and stored at −80 °C before being shipped on dry ice for global metabolomic analysis at Metabolon, Inc. (Morrisville, NC, USA).

The methodology outlined herein describes the standard procedure employed for analyzing our submitted plasma samples (as provided by Metabolon, Inc.). This protocol, conducted by Metabolon, Inc., was based on their recommendations following plasma metabolome profiling. At Metabolon, each plasma sample was assigned a unique identifier upon receipt to monitor its progress during subsequent processing steps. For analysis, the samples underwent preparation using air displacement pipetting on an automated MicroLab STAR^®^ system by Hamilton Company, ensuring precise measurement accuracy. Quality control measures were implemented by adding recovery standards prior to metabolite extraction. To remove proteins and recover diverse metabolites, plasma proteins were precipitated with methanol through vigorous shaking for two minutes using the Glen Mills GenoGrinder 2000, followed by centrifugation. The resulting extract was fractionated into five parts as follows: two for analysis using separate reverse-phase (RP)/UPLC-MS/MS methods with positive ion mode electrospray ionization (ESI), one for RP/UPLC-MS/MS analysis with negative ion mode ESI, one for HILIC/UPLC-MS/MS analysis with negative ion mode ESI, and one backup sample. Subsequently, samples were briefly treated with a TurboVap^®^ (Zymark) to eliminate organic solvent residues before being stored under nitrogen overnight in preparation for analysis. Before analyzing the samples, specific controls were run through the instrument to ensure its performance and to achieve proper chromatographic peak alignment, thus ensuring the quality of analysis results.

### 2.3. Ultra-High-Performance Liquid Chromatography/Tandem Accurate Mass Spectrometry (UHPLC/MS/MS)

All methods employed a Waters ACQUITY ultra-performance liquid chromatography (UPLC) system and a Thermo Scientific Q-Exactive high-resolution/accurate mass spectrometer. These instruments were equipped with a heated electrospray ionization (HESI-II) source and Orbitrap mass analyzer operating at a mass resolution of 35,000. The sample extract was dried and then reconstituted in solvents tailored for each of the four methods. To ensure consistency in injection and chromatography, each reconstitution solvent included a set of standards at fixed concentrations.

One portion of the sample was subjected to analysis under acidic positive ion conditions, optimized for more hydrophilic compounds. This involved gradient elution from a C18 column using a mixture of water and methanol. Another portion underwent analysis under similar acidic positive ion conditions but was optimized for hydrophobic compounds, with a higher overall organic content in the eluent. A third portion was analyzed under basic negative ion conditions using a dedicated C18 column, with gradient elution using methanol and water containing ammonium bicarbonate. The fourth portion was analyzed via negative ionization after elution from a HILIC column using a gradient of water and acetonitrile with ammonium formate. The mass spectrometry analysis alternated between MS and data-dependent MSn scans with dynamic exclusion. The scan range varied slightly between methods but typically covered 70–1000 m/z. Raw data files were archived and processed.

The bioinformatics system comprised four key components including the following: the Laboratory Information Management System (LIMS), data extraction and peak-identification software, tools for data processing and compound identification, and information interpretation and visualization tools. These components were supported by a LAN backbone and an Oracle 10.2.0.1 Enterprise Edition database server. The Metabolon LIMS enabled comprehensive and auditable laboratory automation, covering sample accessioning, preparation, instrumental analysis, and reporting. The system served as the foundation for subsequent software systems and was modified to interface with in-house and third-party instrumentation and software.

### 2.4. Data Extraction and Compound Identification

Utilizing Metabolon’s software and hardware, the raw data underwent extraction, peak identification, and quality control processes. Compounds were identified by comparing them to entries in a library of purified standards or recurring unknown entities. Metabolon maintains an updated library containing retention time/index (RI), the mass-to-charge ratio (*m*/*z*), and chromatographic data on all molecules. Biochemical identifications were based on the following three criteria: retention index, accurate mass match, and MS/MS scores. Additionally, more than 3300 purified standard compounds were registered into LIMS for analysis, and additional mass spectral entries were created for structurally unnamed biochemicals identified by their recurrent nature. The curation process ensured the accurate identification of true chemical entities and the removal of artifacts, mis-assignments, and background noise. Metabolon’s analysts used proprietary software to confirm peak consistency among samples and checked library matches for each compound in every sample, making corrections if necessary.

### 2.5. Metabolite Quantification and Data Normalization

To quantify peaks, we employed the area under the curve method. To address variations caused by differences in instrument tuning among days in studies spanning multiple days, a data normalization step was undertaken. This process essentially equalized the medians of each compound to one (1.00) within run-day blocks and normalized each data point proportionately (known as the “block correction”). However, studies conducted within a single day did not require normalization, except for data visualization purposes.

### 2.6. Metabolomics Analysis

The generated data contained metabolites with both known and unknown biochemical identities. The preprocessed output data consisted of tables populated with normalized peak intensity values for each metabolite across all plasma samples. These data were exported to MetaboAnalyst 5.0 (Version 5.0, McGill University; Montreal, QC, Canada) for univariate analysis. Prior to submitting the input file (csv format) to the software, a data inspection was conducted to identify and average any duplicate values for each metabolite to prevent errors during the integrity check of the input data. Subsequently, skewed data were transformed to achieve a Gaussian normal distribution through log normalization to address heteroscedasticity. Additionally, the data were autoscaled, setting the standard deviation to 1, enabling analysis using correlations instead of covariances. Following the univariate analysis, the output data included a fold change data file, which could be visualized using volcano plots to aid in identifying and discriminating altered metabolites between JIA and non-JIA patients.

### 2.7. Enrichment Analysis

Enrichment analysis was conducted using fold change and *p*-values obtained from MetaboAnalyst 5.0 output. Chemometric analysis utilized the open-source software, ChemRICH (University of California, Davis, CA, USA) for Chemical Similarity Enrichment Analysis for Metabolomics, which leverages structural similarities and chemical ontologies to generate nonoverlapping sets of recognized metabolites. In the analysis, compounds were mapped to MeSH terms using the NCBI resource, while substructure fingerprints from PubChem resources were used to calculate Tanimoto chemical similarity coefficients. These coefficients were then used to generate a pairwise similarity matrix. The Tanimoto calculation formula, T (A, B) = AB/(A + B) − AB, was applied to determine the similarity between compounds A and B based on their substructure bits. The output data were represented by a bubble chart, visually clustering metabolites into groups based on *p*-values (*p* < 0.05). Chemometric and biochemical network maps were visualized using MetaMapp 2020 (University of California, Davis, CA, USA), constructed with Tanimoto substructure similarity coefficients, and the Kyoto Encyclopedia of Genes and Genomes (KEGG) metabolic network database. The processed data were displayed using Cytoscape 3.9.1 (Version 3.9.1, Institute for Systems Biology, Seattle, Washington, DC, USA). Furthermore, univariate receiver operating characteristic (ROC) curve analysis was performed in MetaboAnalyst 5.0 to calculate the area under the curve (AUC). Metabolites with AUC > 0.90 were identified as highly discriminating biomarkers between diseased and healthy samples.

### 2.8. Statistical Analysis

Patient data for age are presented as mean and standard deviation (mean (SD)). Differences between groups were tested using the two-tailed Mann–Whitney U test. Prior to this, the Shapiro–Wilk test was conducted to assess the normality of the data. Subsequently, differences in gender composition between the cohorts were evaluated by Fisher’s exact test. The chosen significance level of 0.05 (α = 0.05) was applied universally across all statistical analyses.

The significance threshold was predetermined as a false discovery rate-adjusted *p*-value (*q*-value) less than 0.5 (*q* < 0.05) and was employed for metabolomic analysis across discovery, replication, and merged datasets. Chemometric analysis utilized ChemRICH, with significant metabolites defined by a *q*-value of less than 0.05. ROC curve plots were generated with an area under the curve (AUC) threshold of 0.90. The plots were created using GraphPad Prism 8.0.1.

## 3. Results

### 3.1. Demographic Data for Discovery and Replication Cohorts

For the analysis in this study, only age and gender were considered for demographics. In the analysis, age emerged as a significant parameter at baseline, with the patient population exhibiting slightly older ages in the non-JIA group, as indicated by the *p*-value (for discovery *p* = 0.027, and for replication *p* = 3.8 × 10^−4^) and summarized in Table 1.

In addition, the race distribution of the JIA cohorts is as follows: In the discovery cohort (*n* = 60), there were 36 White individuals, 1 Black, African American, African, or Afro-Caribbean individual, and 1 White individual who reported Hispanic, Latino, or Spanish origin ethnicity, and data for 23 patients were not recorded or available. In the replication cohorts (*n* = 49), there were 47 White individuals, 1 Black, African American, African, or Afro-Caribbean individual; 2 of the White individuals reported Hispanic, Latino, or Spanish origin ethnicity, and data for 1 patient was not recorded or available. Race/ethnicity data were not recorded or available for the non-JIA cohorts; therefore, ethnicity was only reported for one group and was not included in the analysis.

### 3.2. Metabolomic Profiling to Identify Plasma Metabolites Altered in Children with JIA

**Figure 2:**
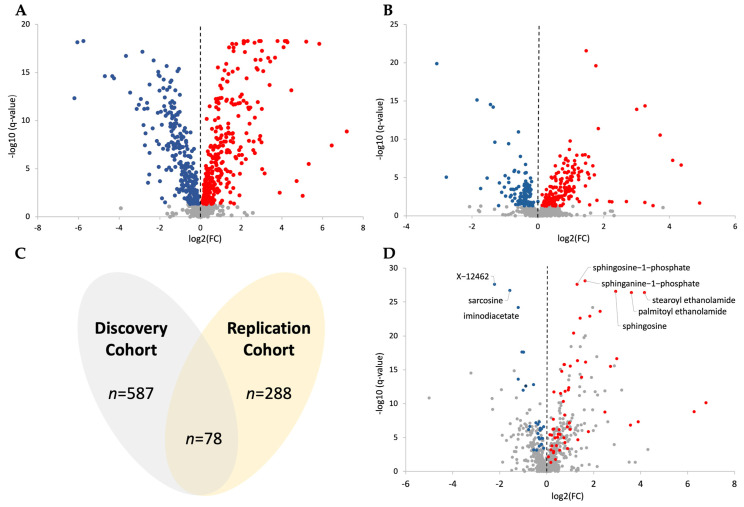
The analyzed metabolomic data represented graphically. (**A**) Discovery dataset volcano plot. (**B**) Replication dataset volcano plot. (**C**) Venn diagram for the significant data (*q*-value < 0.05) for both datasets, where the overlapped portion represents the merged dataset. (**D**) Merged dataset volcano plot. The volcano plot color scheme is as follows: red (upregulated), blue (downregulated), and grey are all non-significant data points.

Plasma samples were submitted to Metabolon for global metabolic profiling as two separate batches. In the discovery analysis (Figure 2A), a total of 906 metabolites were identified, with 587 metabolites showing significant differences (*q* < 0.05) between patients with JIA and those without (non-JIA). In the replication analysis (Figure 2B), a total of 863 metabolites were identified with 288 significantly altered (*q* < 0.05). When only metabolites identified as significantly different in both analyses were considered, it was found that 78 metabolites overlapped with the same directional trend in both datasets (Figure 2C). Data from both analyses were combined into one merged dataset for analysis. The data from this merged analysis are presented in a separate volcano plot (Figure 2D). In this volcano plot, metabolites are colored only if they (1) achieved a statistical significance threshold in the merged analysis (*q* < 0.5), (2) were significantly different in both the discovery and replication analysis (*q* < 0.5), and (3) demonstrated the same directional trend in both analyses (i.e., upregulated or downregulated). Based on the merged analysis, JIA was associated with differences in plasma levels of various metabolites including an increase in several sphingosine metabolites and fatty acid ethanolamides. Similarly, JIA was associated with decreased plasma levels of various metabolites including sarcosine, iminodiacetate, and the unknown metabolite X-12462 (Figure 2D). 

### 3.3. Chemical Enrichment Analysis and Network Visualization to Identify Metabolic Alterations in JIA

Further, we used ChemRICH to categorize known metabolites and identify metabolic clusters altered in patients with JIA. This tool employs medical subject heading (MeSH) annotations and Tanimoto substructure chemical similarity coefficients to group metabolites into distinct chemical clusters.

The bubble plot presents data on the following two key parameters: fractional directional change and XlogP value. Fractional directional change quantifies the net change in metabolite levels relative to the total number of measured metabolites in each group. Meanwhile, the XlogP value, derived from the log of the partition coefficient between n-octanol and water, is an important chemical descriptor used to assess metabolite lipophilicity in quantitative structure–activity relationship (QSAR) studies.

**Figure 3:**
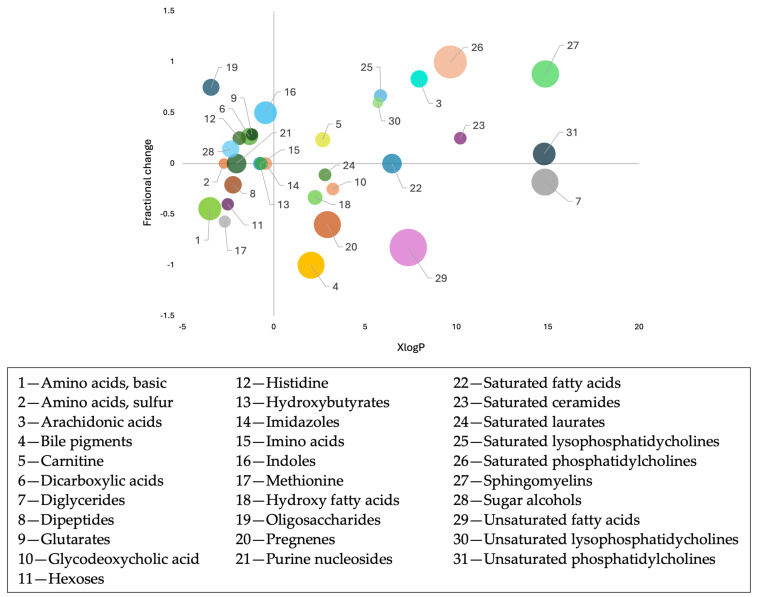
Representation of ChemRICH enrichment analysis as a bubble plot of the annotated metabolites from the merged global metabolomic analysis. The analysis uses chemical similarity and ontology mapping to generate metabolite clusters. Each node represents a metabolite cluster that significantly differs in patients with JIA and the q-value of each metabolite cluster is represented by the size of the node. The *y*-axis provides the fractional change for each class of metabolites, and the *x*-axis provides the XlogP as a chemical descriptor, facilitating the stratification of metabolites based on their lipophilicity properties. The colors in the graph are used solely to represent different clusters.

To summarize, the fractional directional change indicates the overall shift observed across a cluster of metabolite data points. A predominant increase or decrease in metabolite levels within this cluster suggests a directional trend. Additionally, the node size in the plot corresponds to the negative log-transformed false discovery rate-adjusted *p*-value (*q*-value) for each metabolite class. The chemometric enrichment analysis aided in revealing identified metabolites grouped in 31 annotated clusters (Figure 3). The top discriminating biochemical clusters with positive fractional change were observed in different classes of sphingolipids as well as in saturated phosphatidylcholines. Similarly, significant metabolites with negative fractional change were observed for bile pigments and unsaturated fatty acids, whereas saturated fatty acids did not exhibit large negative fractional change.

Following this, MetaMapp was employed for efficient mapping and visualization of metabolomic data, integrating information from biochemical pathways and chemical and mass spectral similarity. It mapped detected metabolites into network graphs using the KEGG reactant pair database and Tanimoto chemical/NIST mass spectral similarity scores. Compared with conventional biochemical mapping, MetaMapp graphs in Cytoscape provide clearer metabolic modularity and comprehensive content visualization.

**Figure 4:**
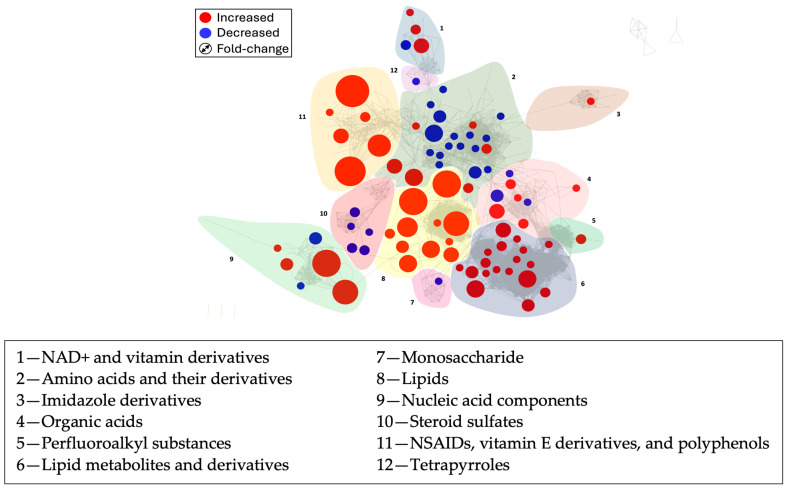
MetaMapp metabolite network visualization in Cytoscape 3.9.1 open-source software. The red nodes represent increased metabolites in JIA compared with the control, while the blue nodes represent a decrease. The size of the node indicates the magnitude of the fold change. This is illustrated in the upper left box, specifically represented by the Fold-change symbol. These compounds are connected by chemical similarity and KEGG reaction pairs. Here, NSAIDs refer to Nonsteroidal Anti-Inflammatory Drugs. Additionally, the figure highlights two distinct lipid categories as follows: lipids (6), which includes acylcarnitines, glycerophospholipids, cholines, and fatty acids, and lipids (8), comprising sphingolipids, phospholipids, and fatty acid amides.

The results yielded a network map illustrating the connection between metabolites and their respective pathways (Figure 4). In this visualization, node size correlates with the observed median fold change, depicting the relative differences in metabolite abundance between experimental conditions, while nodes are color-coded to indicate whether the metabolite was upregulated (red) or downregulated (blue). Further classification of resulting node clusters was performed manually based on the chemical composition and associated metabolic pathways of the metabolites within each cluster. Noteworthy, upregulated clusters included lipid metabolites and derivatives (cluster 6), comprising acylcarnitines, glycerophospholipids, cholines, and fatty acids, as well as lipids (cluster 8) containing sphingolipids, phospholipids, and fatty acid amides. Other upregulated clusters involved nucleic acid components (cluster 9), and a cluster (cluster 11) consisting of NSAIDs (including naproxens), vitamin E derivatives, and polyphenol metabolites. In contrast, the downregulated clusters primarily included amino acids and their derivatives (cluster 2) and steroid sulfates (cluster 10).

### 3.4. Identification of Putative Small-Molecule Biomarkers of Disease in JIA

To assess the predictive value of individual metabolites, we conducted ROC analysis. Using MetaboAnalyst, univariate ROC curve analysis revealed several plasma metabolites with high AUC values exceeding 0.9, indicating strong discriminatory potential as diagnostic biomarkers for the disease.

Notably, our ROC analysis focused on metabolites significantly altered in both the discovery and replication datasets, exhibiting consistent regulatory trends (i.e., upregulated or downregulated) across the datasets. Subsequently, the merged ROC data were compared, highlighting metabolites consistently showing significance and consistent regulatory trends across all three datasets.

**Figure 5:**
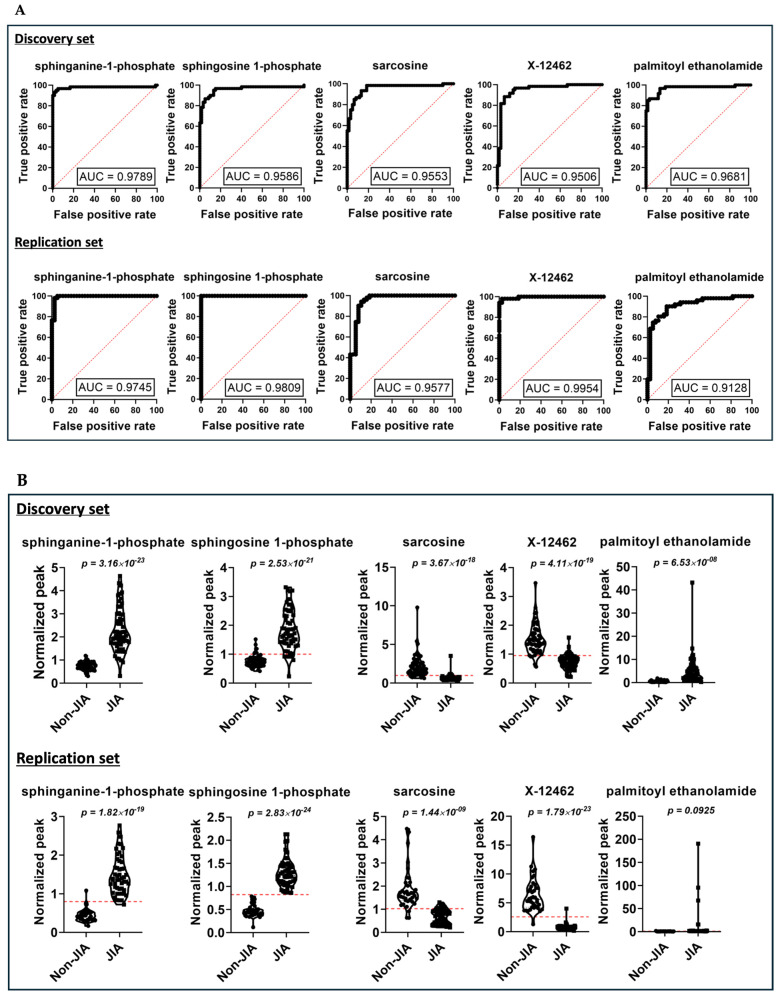
ROC univariate analysis revealing the top five discriminating metabolites based on the threshold (AUC > 0.9) in the merged dataset. (**A**) The receiver operating characteristics (ROC) curve analysis for both the discovery and replication datasets. The solid black line represents the ROC curve for each individual metabolite, whereas the red dotted line represents the line of no discrimination, i.e., it does not have any significant diagnostic or prognostic value. For analysis, the Wilson and Brown hybrid method was used with a confidence interval of 95%, leading to the binomial distribution of the dataset. The *x*-axis, i.e., false positive rate represents 100%—Specificity%, whereas the *y*-axis, i.e., true positive rate represents Sensitivity%. (**B**) The violin plots from both datasets represent the normalized distribution of plasma metabolite sample features. The data points (solid circles and solid squares) represent the concentration of the selected features from all samples. The red dotted lines in the violin plots indicate the “optimal cutoff” threshold, maximizing sensitivity and specificity for a diagnostic test. In cases where the cutoff line is absent, it suggests substantial overlap between the two groups or insufficient separation to determine a clear cutoff value.

Following comparative analysis, we identified five biomarkers (Figure 5), including sphinganine-1-phosphate, sphingosine-1-phosphate, sarcosine palmitoyl ethanolamide, and unknown metabolite X-12462. These biomarkers were consistently identified across both the discovery and replication datasets, with sphinganine-1-phosphate emerging as the top discriminatory metabolite, even when the datasets were analyzed separately and as a merged dataset.

## 4. Discussion

In this study, we conducted an untargeted metabolomics analysis on plasma samples obtained from patients diagnosed with JIA at baseline, i.e., prior to initiating DMARD therapy. These samples were compared to those from controls (or non-JIA cohorts not exposed to systemic immunomodulators, immunosuppressants, or biologicals) using UHPLC-MS/MS. The primary objective was to identify circulating metabolomic biomarkers that could potentially improve the early detection and characterization of JIA in a minimally invasive manner (utilizing plasma samples).

Limited literature exists on the metabolomic profile of plasma samples from JIA patients within the population. Therefore, our study aimed to identify endogenous small molecule metabolites in JIA plasma to better understand the overall metabolic changes associated with the disease, aiding in disease diagnosis through the discovery of potential JIA-related biomarkers. We identified five metabolites closely associated with JIA diagnosis, with sphinganine-1-phosphate emerging as the most distinct biomarker. Additionally, X-12462 (unknown metabolite), sphingosine-1-phosphate, palmitoyl ethanolamide, and sarcosine showed promise as potential biomarkers for JIA diagnosis. These metabolites participate in a range of metabolic pathways, including sphingolipid metabolism, cell growth regulation, immune responses, neuroprotection, and inflammation, which are consistent with the current understanding of JIA pathophysiology and suggest their potential role as diagnostic biomarkers [29,30,31,32,33,34,35,36,37,38,39,40,41,42]. Alterations in these metabolites may exert diverse effects on overall metabolism within the body.

Sphingosine, a metabolite of sphingolipids, acts as a structural component within cell membranes while also playing a significant role in signal transduction. This metabolite regulates essential cellular functions such as survival, proliferation, migration, and neovascularization [43,44,45].

Sphingolipids are essential components of cell membranes, serving important functions in cellular signaling and regulation. Notably, sphingosine-1-phosphate (S1P) and sphinganine-1-phosphate (SA1P), among the diverse array of sphingolipids, are recognized as bioactive lipid molecules with profound involvement in numerous physiological and pathological processes, including immune responses and inflammation [34,35,36,46].

**Figure 6:**
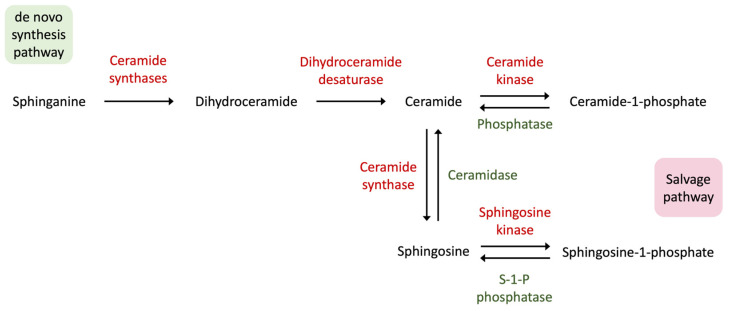
Diagram illustrating the formation of ceramide via both de novo synthesis and the salvage pathway. Ceramide formation through the salvage pathway involves the breakdown of sphingosine. SA1P is primarily synthesized via the de novo pathway. This pathway leads to the formation of ceramide, which can be converted to S1P. In the salvage pathway, SA1P can be indirectly generated from S1P through ceramide breakdown. Here, all the forward reactions are colored in red, whereas the reverse reactions are colored in green. Created in BioRender.com.

The metabolism of sphingolipids, which include important molecules like S1P and SA1P, is closely linked with ceramides, forming a complex network of cellular signaling pathways [34,46,47,48]. Ceramides act as building blocks for S1P and SA1P production, mainly regulated by enzymes like sphingosine kinase and ceramide kinase (Figure 6) [49]. Ceramides are strongly associated with inflammation and cell death, contributing to conditions such as cartilage disorders [50].

However, the specific targets of intracellular S1P are not fully understood, requiring further investigation into their roles in cellular processes. Dysregulation of ceramide metabolism and changes in the balance between ceramides and other active sphingolipids, including S1P and SA1P, have been linked to the development of inflammatory diseases like arthritis, which further emphasizes the need to understand their complex relationship in inflammatory processes and autoimmune conditions such as JIA [49,50,51].

**Figure 7:**
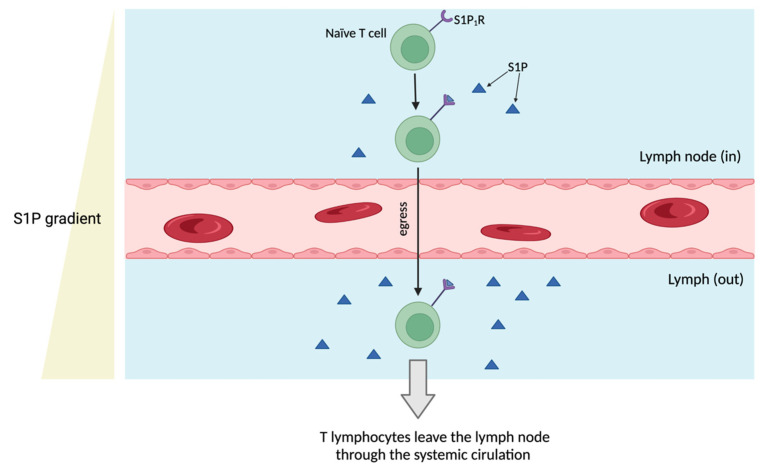
Sphingosine-1-phosphate: Orchestrating lymphocyte egress and immune response. This complex signaling pathway, crucial for the movement of lymphocytes and the stability of blood vessels, plays a critical role in regulating intricate inflammatory responses. Remarkably, S1P is highly concentrated in blood and lymphatic fluids, while remaining at low levels within cells or in the spaces between cells. This characteristic creates a significant S1P gradient necessary for efficiently guiding lymphocytes out of lymphoid organs. The components in the figure include a naïve T cell with S1P receptor (S1P1R), a lymph node (in), a lymphatic vessel (out), an S1P gradient, and a blood vessel (red), illustrating the pathway involved in lymphocyte egress. Created using BioRender.com.

Alexandropoulou et al. observed increased levels of ceramides, including d18:1/24:2, d18:1/24:0, and S1P, in inflammatory conditions [49]. This trend in sphingolipid upregulation, including S1P and SA1P, was also observed in our output data. Considering S1P’s role in regulating inflammation and immune responses via receptor binding, and its impact on cellular growth and apoptosis, targeting both S1P and its receptor (S1PR) shows therapeutic potential in autoimmune arthritis, as evidenced in RA [52,53]. For example, S1P governs cellular egress through interaction with lymphocyte receptors, orchestrating intracellular signaling pathways that guide lymphocyte migration from lymphoid reservoirs to the circulatory system. This process establishes an S1P concentration gradient, with higher levels in blood and lymphatic fluids and lower levels in tissues, directing lymphocytes away from lymphoid organs and modulating immune surveillance and response mechanisms (Figure 7) [52,54].

Furthermore, studies conducted in vitro have revealed the involvement of ceramides in programmed cell death in RA [49]. Additionally, changes in sphingolipids, particularly elevated ceramide levels, have been identified in the synovial fluid of patients with osteoarthritis (OA) and RA compared with controls, suggesting potential implications for clinical interventions in joint disorders [55,56,57]. Clinical treatments like methotrexate have demonstrated efficacy in normalizing plasma ceramide levels, providing valuable insights into potential therapeutic strategies for arthritis management [58]. Furthermore, animal studies have highlighted the effectiveness of targeting sphingolipids as a viable approach for alleviating arthritis symptoms and reducing circulating inflammatory mediators, suggesting the possibility of novel therapeutic interventions in arthritis management [59,60,61,62,63].

Discussing the need for diagnostic biomarkers of JIA highlights the challenges associated with patient misdiagnosis. One classic example comes from Farber Disease (FD), where misdiagnosis commonly arises because of symptom overlap between JIA and FD, complicating identification. Additionally, the variability in responses to treatments for JIA can further complicate achieving an accurate diagnosis, emphasizing the urgent requirement for new biomarkers to distinguish between these conditions or at least for JIA [64,65,66,67]. One previous study revealed alterations in cytokine and ceramide levels in FD patients, aiding in understanding the disease’s mechanisms and differentiating it from JIA [68]. However, while certain cytokines (Interleukins) and ceramides (C20:1-Cer, dhC18-Cer) serve as useful markers, their limited utility underscores the pressing need for additional biomarkers to enhance diagnostic precision.

In addition to sphingolipids, two other important metabolites identified in our study were palmitoyl ethanolamide and sarcosine. Palmitoylethanolamide (PEA) has been extensively researched for its ability to reduce pain and inflammation, particularly in OA [69]. Evidence from multiple studies and trials supports its effectiveness in relieving pain in OA patients, although the exact mechanisms involved require further investigation [69,70,71,72]. Experimental models of OA have shown that PEA can help prevent cartilage degradation and reduce joint swelling by lowering inflammation and inhibiting enzymes that damage cartilage [73].

Similarly, in a study using an arthritis model (collagen-induced arthritis model), researchers examined urine metabolites in arthritic mice, aiming to identify biomarkers for muscle loss. They found significant changes in muscle-related metabolites and pathways, with sarcosine exerting notable effects throughout various disease stages [74]. In a separate biomarker study aimed at identifying early diagnostic markers for RA, sarcosine was found to be reduced in the serum of individuals with early-stage rheumatoid arthritis [75] We also observed a similar downward trend in sarcosine levels in our study.

Existing data on the effects of PEA and sarcosine in conditions like JIA are either absent or limited. However, our study is the first to explore a potential link among PEA, sarcosine, and JIA. Our research revealed an upregulation of PEA and observed opposite trends for sarcosine within this JIA cohort, suggesting their relevance as diagnostic indicators for JIA. Finally, although the compound X-12462 remains unidentified, preliminary discussions with Metabolon Inc. (unpublished) suggest its potential structure as C5H9NO2S, hypothesized to be vinyl cysteine, although this remains uncertain until further confirmation.

This study has several limitations. Firstly, the relatively small sample size may affect the robustness of the findings, especially considering the extensive range of metabolites analyzed. For missing values in the untargeted metabolomics dataset, we used batch-normalized imputed data, where values below the limit of detection were replaced with the observed minimum for each metabolite post-normalization to reduce false negatives and enhance statistical power. The samples were collected from different cohorts using similar preservatives and collection methods, but the procedures were not conducted in the same laboratory or by the same personnel, introducing covariates that cannot be fully controlled in clinical studies. Additionally, variations in the fed/fasting state across study cohorts could influence metabolite levels. Specifically, all samples (non-JIA) from the CM-KC repository were collected in the morning (7:30–11:30 A.M.) in a fasted state, which might differ from other cohorts and potentially affect endogenous concentrations because of diurnal patterns. We also lacked information on the JIA subtypes included in the study. Furthermore, the potential influence of concomitant medications on metabolite concentrations was not controlled for, which may introduce variability. Another limitation is the combination of Crohn’s disease and Functional Abdominal Pain cohorts into a single non-JIA cohort. This approach was chosen to compare JIA against a composite non-JIA group, including both Crohn’s patients and healthy controls, but it could complicate data interpretation. Clearly articulating the rationale for this comparison is essential for understanding its implications on this study’s outcomes.

Moreover, in untargeted metabolomics studies, the challenge lies in managing a large number of differential metabolites. Reliable reporting of results is jeopardized when low-sensitivity or low-quality integrations from untargeted UHPLC-MS/MS are not eliminated [76]. Thus, optimizing data quality control conditions is crucial to ensure thorough and precise information about changes in metabolites. Additionally, involving a larger number of patients in the study adds complexity, but testing outcomes with larger cohorts can yield more distinct metabolites and validate previously identified ones, enhancing a study’s outcomes [77].

Metabolomics, genomics, and proteomics are interrelated research fields that demand coordination throughout the research process. Integrating the findings of metabolomics with genomics, proteomics, and other studies enables a comprehensive examination of a patient’s overall condition, shedding light on changes before and after the onset of diseases like JIA from a systemic biology standpoint [78,79,80].

## 5. Conclusions

In this study, we performed untargeted metabolomics profiling on plasma samples from two (discovery and replication) cohorts. Analysis of the combined dataset revealed five distinct metabolites including sphinganine-1-phosphate, X-12462, sphingosine-1-phosphate, palmitoyl ethanolamide, and iminodiacetate. These metabolites hold potential as biomarkers for diagnosing patients with JIA or uncovering underlying pathophysiologic mechanisms. In particular, our findings suggest that sphingosine-1-phosphate (S1P) and its role in lymphocyte trafficking could be a key mechanism driving the chronic inflammation observed in JIA, contributing to disease persistence in affected joints.

## Figures and Tables

**Table 1:** Baseline demographic data for both cohorts (discovery and replication) are presented in the table. The *p*-value for age was calculated using the Mann–Whitney two-sample *t*-test. The significant values (*p* < 0.05) are highlighted in bold. The data for age are represented as mean (standard deviation), N as the number of subjects, unless otherwise stated.

Baseline	JIA	Non-JIA	JIA vs. Non-JIA (*p*-Value)
Discovery			
Patient, no. (N)	60	60	---
Age (years), mean (SD)	10.15 (4.50)	11.88 (4.45)	**0.027**
Female, N (%)	40 (66.67)	31 (51.67)	0.137
Replication			
Patient, no. (N)	49	38	---
Age (years), mean (SD)	10.35 (4.96)	14.48 (3.02)	**3.8 × 10^−4^**
Female, N (%)	34 (69.39)	20 (52.63)	0.125

## Data Availability

The datasets generated and analyzed during this study are available in the Metabolomics Workbench database (https://www.metabolomicsworkbench.org/, accessed on 28 July 2024), accessible under Project ID 5184.
